# Harnessing Synthetic Ecology for commercial algae production

**DOI:** 10.1038/s41598-019-46135-6

**Published:** 2019-07-05

**Authors:** Sam A. Reynolds, Matthew P. Davey, David C. Aldridge

**Affiliations:** 1 Department of Zoology, University of Cambridge, The David Attenborough Building, Pembroke Street, Cambridge, CB2 3QZ UK; 2 Department of Plant Sciences, University of Cambridge, Downing Street, Cambridge, CB2 3EA UK

**Keywords:** Freshwater ecology, Ecology

## Abstract

Synthetic Ecology is a novel concept describing the design of *de novo* ecological communities for a designated purpose. This study is a proof of concept for harnessing Synthetic Ecology in expanding the scale of commercially relevant micro algae (*Chlorella vulgaris*) cultivation using stable Synthetic Ecologies in open environments as opposed to vulnerable monocultures. We focused on whether the grazing activity of zebra mussels (*Dreissena polymorpha*) would result in a consistent, and commercially favourable, dominance of *Chlorella* in cultures that were also inoculated with a competing and potentially invasive cyanobacteria (*Synechocystis sp. PCC6803*). The key result of this study was that in axenic mixed species co-cultures, zebra mussels had a significantly greater negative effect on *Synechocystis* cell numbers than *Chlorella* (P < 0.0001). The zebra mussels’ putative preference for *Synechocystis* over *Chlorella* suggests they could be used to maintain the dominance of *Chlorella* in outdoor cultivation systems prone to contamination by invasive cyanobacteria.

## Introduction

The goal of synthetic biology is to extend or modify the behaviour of organisms and engineer them to perform new tasks^1^. Simplistically, this is achieved by deconstructing the biology of an organism to a hierarchy of its constituent parts and understanding how the context and interactions between these distinct parts dictates the organism’s phenotype and behaviour. The organism can then be reprogrammed genetically to achieve a desired characteristic or behaviour. Synthetic Ecology aims to apply the same principles to the behaviour and design of artificial ecosystems that could be used to enhance biotechnological processes^2^. Through characterising the hierarchy of ecological interactions in a desired model system, a *de novo* system could theoretically be created through the artificial combination of nutrients and deliberately designed synthetic communities of organisms. This technique could be used to design systems which provide beneficial ecosystem services while behaving as self-regulating climax communities resilient to biological invasion.

The aim of our study was to investigate a proof of concept for the emerging field of Synthetic Ecology, studying the ability of filter feeding zebra mussels (*Dreissena polymorpha*) to drive and stabilise a shift in a *de novo* algal community to enable the proliferation of an algal species of commercial interest (*Chlorella vulgaris*). An alternative to popular high-cost small-scale closed production systems is growing algae in open raceways, which have lower associated construction costs and environmental burdens in terms of energy consumption^3^. However, the current drawback to algae production in open raceways is maintaining the optimum conditions for algae growth, while limiting contamination from invasive organisms which introduce competition and reduce the yields of the target cultivated algae species.

The zebra mussel is a prolific invasive bivalve, originating from the Black, Caspian and Azov Sea regions of central and eastern Europe. Several studies have focused on assessing the impact of the zebra mussel in its invaded range on existing algal communities^4–7^. A single zebra mussel filters approximately two litres of water per day^8^ and significantly reduces the total biovolume of cyanobacteria in reservoir mesocosm experiments^7^. Additionally, Smith *et al*., (1998) measured a shift in dominance from cyanobacterial to diatom species when analysing changes in the cell density and taxonomic composition of phytoplankton assemblages in the freshwater portion of the Hudson River before and after the successful invasion of zebra mussels^5^.

Increases in nutrients, especially phosphorus (P), will stimulate algal production^9^ and algal biomass is tightly linked to phosphorus in lakes worldwide^10^. However, Zebra mussels have been shown to negate or mask any positive growth effects of nutrient enrichment on algal biomass up to 150 µg of phosphorus per litre (Dzialowski & Jessie 2009). It has also been suggested that the effect of zebra mussels on the cyanobacteria *Microcystis spp*. is nutrient dependant^11–13^. Raikow *et al*. (2004) noted a positive relationship between zebra mussels and *Microcystis* spp. in lakes with low Total P concentrations (<0.25 µg/L), but no relationship between zebra mussels and *Microcystis* spp. in lakes with high Total P concentrations (>0.25 µg/L).

This study aimed to distinguish whether zebra mussels under certain nutrient conditions can be utilised to drive a favourable shift in a *de novo* algal co-culture by suppressing the growth of a potentially invasive cyanobacterial species (*Synechocystis sp. PCC6803*) and so supporting the proliferation of a commercially valuable green algae species (*Chlorella vulgaris*). *Chlorella* is a widely cultivated microalgae species and has application as a health food and food supplement, alternative agricultural feedstock, production of biofuels, as well as having many uses in the pharmaceutical and cosmetics industry^14,15^. By demonstrating the ability to achieve desirable end points in this model system this would open the opportunity for developing Synthetic Ecologies in more complex communities and for other applications.

Therefore, the main aims of this study were twofold: (1) investigate the effect of zebra mussels on a simple axenic algal community structure and (2) to identify whether the relationship between zebra mussels and cyanobacteria is dependent on the levels of Total P.

## Results

**Figure 1:**
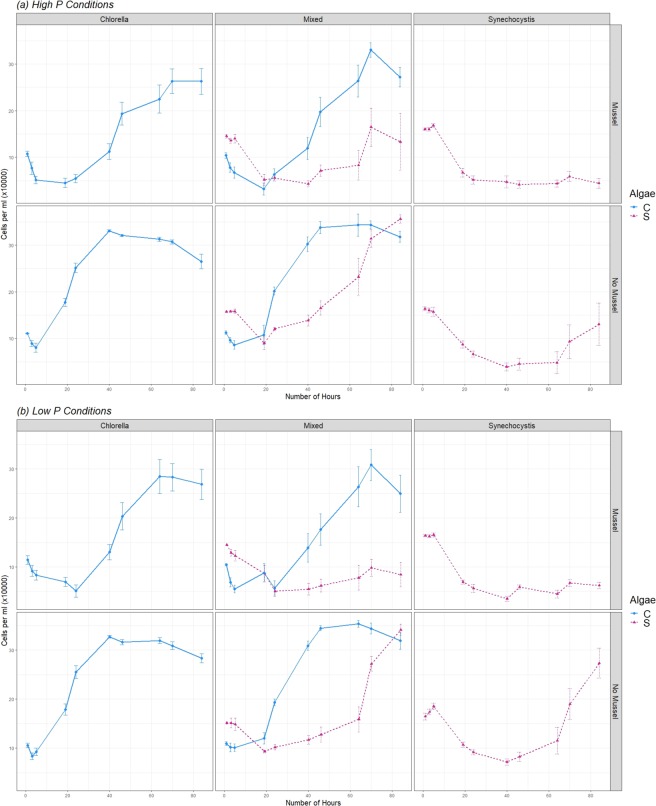
(**a**) Changes in the cell numbers over time of *Chlorella* and *Synechocystis* in single algae and mixed species treatments, in both the presence and absence of mussels, in high P conditions. (**b**) Same condition variations as panel (a), but in Low P conditions. Solid lines and circular points indicate *Chlorella* cell numbers. Dotted lines and triangular points indicate *Synechocystis* cell numbers. The error bars indicate standard error.

The presence of mussels resulted in a significant decline in the number of cells for both species in all treatment conditions when compared to treatments in the absence of mussels (*Chlorella* (Z Value −7.8, P < 0.0001); *Synechocystis* (Z Value −7.1, P < 0.0001)) (Table 1; Table 2; Fig. 1).

**Table 1:** Random effect variance and standard deviation with fixed effect coefficient estimates, standard errors and significance tests for a GLMM (with a Poisson error distribution) of the number of *Chlorella* cells per ml as a function of phosphate (P) level, the presence or absence of mussels, mixed or single species growth treatments, and as a combination of mixed growth treatment and mussel presence.

*Groups*	*Name*	*Variance*	*Std. Dev*.	
**Random Effects**				
Treatment Number	(Intercept)	0.0181	0.1347	
No Hours	(Intercept)	0.2758	0.5252	
Number of obs: 480. Groups: Treatment.Number, 48; No.Hours, 10				
**Fixed Effects**				
	***Estimate***	***Std. Error***	***Z value***	***Pr(*** **>** ***|z|)***
(Intercept)	12.2144	0.1738	70.2870	<0.0001***
P: High P	−0.0333	0.0389	−0.8570	0.3920
Mussel: Mussel	−0.4297	0.0550	−7.8110	<0.0001***
Treatment.Type: Mixed	0.0050	0.0551	0.0910	0.9280
Mussel:Mussel*Treatment.Type:Mixed	0.0067	0.0778	0.0860	0.9310

**Table 2:** Random effect variance and standard deviation with fixed effect coefficient estimates, standard errors and significance tests for a GLMM (with a Poisson error distribution) of the number of *Synechocystis* cells per ml as a function of phosphate (P) level, the presence or absence of mussels, mixed or single species growth treatments, and as a combination of mixed growth treatment and mussel presence.

*Groups*	*Name*	*Variance*	*Std.Dev*.	
**Random Effects**				
Treatment Number	(Intercept)	0.0500	0.2236	
No. Hours	(Intercept)	0.1287	0.3587	
Number of obs: 480. Groups: Treatment.Number, 48; No.Hours, 10				
**Fixed Effects**				
	***Estimate***	***Std. Error***	***Z value***	***Pr(*>*|z|***)
(Intercept)	11.6367	0.1352	86.0670	<0.0001***
P: High P	−0.0642	0.0646	−0.9930	0.3206
Mussel: Mussel	−0.3078	0.0916	−3.3600	0.0008***
Treatment.Type: Mixed	0.4128	0.0916	4.5070	<0.0001***
Mussel:Mussel*Treatment.Type:Mixed	−0.3304	0.1292	−2.5570	0.0106*

In mixed species treatments, *Synechocystis* grew more successfully than when grown in monoculture (Z Value 4.5, P < 0.0001). The growth of *Chlorella* in mixed species co-culture and in monoculture experimental conditions was similar (Table 1). The initial lag phase of *Chlorella* appears to be slightly elongated when grown in a mixed species co-culture treatment, but the stationary phase achieved similar total cell numbers (Fig. 1). Although the presence of mussels did have a significant effect on *Chlorella* cell numbers, the effect was small in comparison to the effect of mussels on the number of *Synechocystis* cells. The growth of *Synechocystis* was negatively affected to a much greater extent when grown in the presence of mussels, both in single species treatments and mixed species treatments (Z value −2.6, P < 0.01).

The initial level of P did not have a significant measurable effect on the number of cells for either species in any of the experimental treatments (Table 1; Table 2).

## Discussion

The key findings of the study are that both *Chlorella* and *Synechocystis* cell numbers were significantly reduced when zebra mussels were present; the presence of mussels in mixed species treatments caused the number of *Synechocystis* cells to drop more than the number of *Chlorella* cells; *Chlorella* cell number was not affected by the presence of *Synechocystis*; *Synechocystis* reached greater cell numbers when grown in a mixed species culture with *Chlorella* than when grown in monoculture; and the initial levels of P had no effect on the cell numbers of either *Chlorella* or *Synechocystis* in the presence or absence of mussels.

### Effect of mussels on cell numbers

The decline of algae and cyanobacteria cell numbers in the presence of zebra mussels is likely due to the filter feeding activity of the mussels. Despite early declines, which are likely due to the algae acclimatising to being removed the stock culture and optimal nutrient conditions, *Chlorella* still reached high densities of 300,000 cells/ml. Zebra mussels have been observed to reduce their filtering activity as food concentration declines, potentially as an energy saving method in response to a reduced food reward to energy expended trade-off^16^. Reduced feeding could also result from satiation of the mussels^16^. Released from grazing pressure and given the optimum conditions for photosynthesis, it may be that the *Chlorella* was able to recover and ultimately divide faster than the zebra mussels were grazing. It has been shown that zebra mussels exhibit feeding rates which are proportional to the amount of algae available, except in extremely high algal concentrations such as those present in this experiment and in algae cultivation systems^17^.

Additionally, zebra mussels were shown to affect the cell numbers of *Synechocystis* to a greater extent than *Chlorella*, which suggests that zebra mussels may express a feeding preference for *Synechocystis* over *Chlorella*. Naddafi *et al*., (2007) illustrated selective feeding by zebra mussels in lake water samples, finding they avoided green algae in preference for cryptophytes, and also that zebra mussels alter their feeding behaviour in relation to algae composition to ingest the phytoplankton with the highest concentrations of long-chain polyunsaturated fatty acids^18^.

This putative preference is interesting as both *Synechocystis* and *Chlorella* are unicellular in morphology and of similar size. At the start of the experiment the average cell size for *Chlorella* was 2.55 µm (s.d ± 0.33) and the average cell size of *Synechocystis sp*. was 2.64 µm (s.d ± 0.38).

Mussels of all sizes, and therefore ages, should be able to graze on these phytoplankton, given their size. Horgan and Mills (1997) showed that phytoplankton morphology did not hamper ingestion rates, with zebra mussels able to ingest unicellular, filamentous and globular colonies indiscriminately^16^. Horgan and Mills (1997) also showed that most particles smaller than the incurrent siphon are subject to grazing^16^. Given that the siphon diameter of a 10 mm mussel, which is significantly smaller than the individuals used in this experiment (35 mm ± 5SE), has a diameter of 2100 µm^19^, both juvenile and adult mussels would have the ability to graze on these phytoplankton.

There is emerging evidence to suggest that *Chlorella*^20^ and some cyanobacterial species^21,22^ will form colonies when under grazing pressure from zooplankton, but even under these circumstances the overall size of the observed colonies (18 cells and 180 µm respectively), remain well within the size ranges of particles drawn through the inhalant siphon of a zebra mussel (<2100 µm^19^) and either digested or deposited as pseudofaeces. Additionally, these colony forming behaviours have not been documented in the presence of mussels. With regards to any potential predator initiated phenotypic plasticity of both *Chlorella* and *Synechocystis*, any changes in individual cell size or colony formation of either phytoplankton species would likely remain within the grazing capacity of zebra mussels, it is unlikely that any changes in this respect would have altered the outcome of the experiment.

### Effect of chlorella on *synechocystis* growth

In the mixed species treatments conducted in both the presence and absence of mussels, *Synechocystis* cell numbers exceeded those observed in monoculture, whereas *Chlorella* achieved similar cell numbers when in a mixed species culture or monoculture. In the absence of mussels *Synechocystis* cell numbers were very similar to *Chlorella* in mixed species treatments by the end of the experiment. Additionally, increases in *Synechocystis* cell number are preceded by increases in *Chlorella*. This may suggest a commensal relationship between *Chlorella* and *Synechocystis* in which *Synechocystis* derives a benefit from *Chlorella* without affecting *Chlorella’s* growth. The literature on the symbioses observed in algae-bacteria co-cultures is extensive, as many micro-algae rely on exogenous sources of cobalamin (Vitamin B_12_), thiamine (Vitamin B_1_) and or biotin (Vitamin B_7_) to grow^23–25^. However, there is a dearth of literature studying symbioses between green algae and cyanobacteria. The suggestion from our study that cyanobacteria may derive nutritional benefits from sympatric algae has considerable implications for understanding ecosystem dynamics in freshwater systems and warrants further investigation.

A further compelling potential explanation for the enhanced cyanobacterial growth in co-cultures lacking mussels is Transgressive Overyielding (TO)^26^. TO is thought to occur when the biomass production of a species community exceeds that produced by either species grown in monoculture, however examples of this are elusive^26^. This theory is an extension from niche theory, which argues that species must use resources in a spatiotemporally complementary manner to ensure co-existence. It may be the case that *Chlorella*-*Synechocystis* co-cultures more efficiently and extensively captured and utilised the available nutrients in the aquaria than *Synechocystis* in monoculture, however because total levels of P and N were not continuously measured, a definitive conclusion on the occurrence of TO cannot be drawn. Additionally, although complementarity may be observed here in allowing *Synechocystis* to contribute to the total biomass in a way that is potentially enhanced by the presence of *Chlorella*, is has been shown TO requires a greater degree of complementarity than simply allows two species to co-exist^27^.

### Effect of high P and low P treatments on algae growth

In contrast to the body of research which demonstrates the ability of zebra mussels to mask the effect of P-enrichment on algal biomass^6,11–13^, this experiment found no significant difference between algal or cyanobacterial biomass grown under different P concentrations, regardless of the presence of mussels (Table 1; Table 2). However, at least for *Chlorella*, this finding aligns with research undertaken by Wong *et al*. (2017) which looked to understand the effect on N and P ratios on biomass and lipid production^28^. Wong *et al*., (2017) observed the highest *Chlorella* biomass production in BBM - N_control_P_limted_ growth media conditions and noted that P did not exhibit a significant effect on biomass production and that P starvation could result in higher amounts of total lipids and positively affect the lipid composition of *Chlorella* cultures^28^. This may suggest that the expected impact of nutrient loading in freshwater systems on algal biomass and the ability of zebra mussels to mask this effect will vary depending on the species of algae present in the system.

### The use of zebra mussels in open raceways

Introduction of zebra mussels to algal raceways could be implemented using meshed cages, as has been suggested for the use of planktivorous fish as biocontrol agents in other studies^29^. This would be the optimal method for simplifying the recovery of mussel biomass from open algal raceways.

As an invasive species, the risks and benefits of encouraging the proliferation of zebra mussels, in either new or established ranges, need to be carefully assessed on a site-by-site basis in order for this application to be used responsibly and successfully. For example, the risk profile varies vastly when comparing the use of zebra mussels in a closed system within an invaded range, to an open system with links to surrounding waterways, in an uninvaded range. Any decision to encourage zebra mussels should only be taken following discussion and appraisal with the relevant authorities at the regional, national or international level, depending on the perceived risks^30^.

It is highly improbable that zebra mussels would be able to proliferate in an algal raceway due to their shallow design. The average depth of algal raceways varies between 0.1–0.3 m^31^, therefore UV radiation exposure from sunlight would be lethal to the larval stages of zebra mussels^32^, for which the lethal dose is 350 mJ/cm^2^. In open water settings, translocated adult zebra mussels can persist in very shallow water due to their protective shells, but settlement of veliger larvae and subsequent recruitment is rarely found at depths <0.5 m due to larval sensitivity to UV radiation^33^.

Although zebra mussels have lower temperature tolerances than many freshwater mussels, studies by McMahon *et al*., (1995) suggest that the minimal incipient upper lethal temperature of the zebra mussel is greater than 30 °C^34^. Serra-Maia *et al*., (2016) estimate the optimal growth temperature of *Chlorella* to be 23.3 °C^35^, and Kessler *et al*., (1985) demonstrated an upper limit of temperature for growth of 14 strains of *Chlorella* in the range of 26 to 30 °C^36^. Therefore, zebra mussels should be able to tolerate most climates suitable for the optimal production of *Chlorella*.

There has been increased attention on the functional role of secondary metabolites produced by phytoplankton as anti-herbivore defences^37^. These chemical defence responses include oxidative bursts, the halogenation of molecules, and the synthesis of secondary metabolites such as polyphenols^38^. We recognise that in order to initiate these defensive responses there will be a metabolic cost which may affect the overall biochemistry, and therefore potentially the protein or lipid content of the phytoplankton. There are some studies which attempt to identify biochemical change in phytoplankton as the result of producing secondary metabolites in response to certain biotic threats, including endophytes^39^, bacteria^40^ and zooplankton^41^ however there are no studies which attempt to look at biochemical changes in response to mussels. Secondary metabolites exhibit both spatial and temporal variability and most are likely to have multiple additional functions, such as anti-fouling^42^ and UV screening^43^. This makes interpreting the presence, composition or levels of secondary metabolites specifically produced as a response to predation, and therefore any potential or predictable biochemical impact they may have, a challenge. However, this is an area of research which may warrant further investigation.

### Applications in synthetically engineered ecosystems for algae production

The zebra mussels’ putative preference for *Synechocystis* over *Chlorella* could have useful application in the field of Synthetic Ecology and in the mass production of algae. Understanding the constraints of this interaction will be key in designing *de novo* communities that could be used to expand the commercial production of algae, such as *Chlorella*, in outdoor production systems where one constraint is competitive suppression by other algae species. The results of this experiment suggest that zebra mussels, at the right density, could dampen the ability of an invasive cyanobacteria to proliferate and bloom further in a *Chlorella* raceway pond system. This is due to the mussels appearing to express a feeding preference for the cyanobacteria over the green algae. Designing systems that can stabilise in a predictable manner is a key concept in Synthetic Ecology. Effective *de novo* ecosystems would enable industrial production of algae, and other crops, to move away from vulnerable mono-cultures by designing resilient poly-culture communities, utilising organisms from different trophic levels, which can produce similar yields with reduced inputs and less direct management.

In addition to the production of certain species of microalgae in open raceways, zebra mussels may have utility in the production of commercially relevant filamentous macroalgal species. Lawton *et al*., (2017) identify the cosmopolitan freshwater macroalgae genus *Oedogonium* as an ideal candidate for open pond systems, as it is resilient, competitively dominant and has biomass productivities comparable with microalgae^44^. Sakharova *et al*., (2018) have illustrated that *Oedogonium* is resistant to the grazing of zebra mussels^45^ and so therefore a coculture system in which zebra mussels remove competing microalgae species, reducing competition for light and nutrients, may help to improve *Oedogonium* yields further.

The methodology used in this research provides a useful template for future work on Synthetic Ecology. For example, contrary to the effects observed in this experiment with *Synechocystis*, many studies have linked the invasion of zebra mussels with the promotion and bloom formation of the toxic cyanobacteria *Microcystis aeruginosa* in the temperate lakes of North America^46,47^. However, more recent studies contradict such findings, indicating that nutrients and temperature are more likely causal factors^11,48^. Studies which rely on field-based observations make it challenging to identify key drivers of community change because so many interacting factors can be at play. By following the methodology used here, using axenic algal communities and creating these systems *de novo* in isolation, may help to elucidate the true effect of zebra mussels on blooms of this harmful cyanobacteria. Accurate characterisation of species relationships and context dependencies will be key in the application of Synthetic Ecology.

A further potential application of synthetic ecologies outside of industrial biomass production could be phycoremediation, which focuses on waste water remediation using algae species. Traditionally research into waste water remediation has focussed on using green algae^49^ and cyanobacteria^50^ in monoculture to sequester excess nutrients such as nitrogen, phosphorus and ammonia. However the field of phycoremediation is quickly realising that co-cultures are both more effective at sequestering nutrients and inherently more stable systems^51^. *Chlorella* has been demonstrated to absorb 45–97% of nitrogen, 28–96% of Phosphorus and reduce the chemical oxygen demand by 61–86% from different types of wastewater including agricultural and sewage^51–55^. Subsequently there is potential to use mussels to remove these nutrient-accumulating micro algae species, ultimately repackaging and removing excess nutrients as mussel biomass. There has already been significant interest in utilising zebra mussels as tools for managing nuisance algae and cyanobacteria in drinking water reservoirs where the mussel has already established^30^ as well as utilising the resultant mussel biomass as a supplement for commercial chicken feed^56^. The field of phycoremediation could present further applications for zebra mussels. The pursuit of Synthetic Ecology in understanding and modelling the governing environmental intricacies and interspecies relationships of algal communities should allow for the creation of evermore stable and predictable *de novo* communities with utility in industrial applications, from commercially significant algae production to phycoremediation and biofuel production.

## Materials and Methods

### Cultivating algae species

The cyanobacteria *Synechocystis sp*. PCC6803 (hereafter *Synechocystis*) (Strain 6803 Wild Type, Culture Collection of Algae and Protozoa, Oban, UK) and green alga *Chlorella vulgaris* (hereafter *Chlorella*) (Strain 211/11B Culture Collection of Algae and Protozoa, Oban, UK) were selected for this experiment. To maximise the growth rate of these algae species they were grown in their prescribed optimal growth media at stock concentrations prior to the experiment.

*Chlorella* was cultured in sterile 3N-BBM-V growth media (25 g/L NaNO_3_; 2.5 g/L CaCl_2_.2H_2_O; 7.6 g/L MgSO_4_.7H_2_O; 7.5 g/L K_2_HPO_4_.3.H_2_O; 17.5 g/L KH_2_PO_4_; 2.5 g/L NaCl) and trace element solution (The following minerals were added to 1 L of DI water in the following sequence: 97 mg FeCl_3_.6H_2_O; 41 mg MnCl_2_.4H_2_O; 5 mg ZnCl_2_; 2 mg CoCl_2_.6H_2_O; 4 mg Na_2_MoO_4_.2H_2_O). Details at (www.ccap.ac.uk). *Synechocystis* was grown in BG11 media (Sigma-Aldrich Cyanobacteria BG11 Freshwater Solution, Dorset, England).

### Zebra mussels and water collection

Zebra mussels were collected from Roswell Pits Site of Special Scientific Interest, Ely, SE England (52.4013° N, 0.2852° E). This lake is a disused, flooded gravel pit disconnected from the adjacent gravel pits and the nearby river Great Ouse. The average length of the mussels used in the experiment was 35 mm ± 5 SE. Twenty-five litres of reservoir water was also collected from Roswell Pits. The water was filtered through a polyethersulfone filter vacuum with a 0.22 µm membrane to remove any algae or bacteria in the sample prior to use in the experiment.

### Experimental design

Experiments were run in one litre capacity aquaria (129 mm ×133 mm ×133 mm) inside a low light laboratory. Each aquarium was filled to a total volume of 250 ml, which included filtered reservoir water and the algae inoculum. The aquaria were first filled with the allocated volume of water (Table 3). The required volume of algae culture was added to the aquaria using a syringe attached to a 4 mm hose. The algae inoculum was removed directly from the stock culture, during its growth phase, prior to being introduced to the aquaria. A LED lighting unit (Mithril Technology Ltd., Surrey, UK) was fitted 20 cm above the aquaria, emitting light 24 hours per day at specific wavelengths (370 nm, 470 nm, 525 nm, 570 nm, 590 nm and 610 nm) to optimise conditions for photosynthesis. The positioning of the aquaria was randomised to eliminate the effect of differential lighting on algal growth in different treatments. The average Photosynthetically Active Radiation (PAR) level was 26 µmol∙m^−2^∙s^−1^. Each aquarium had a constant air supply supplied through a 4 mm internal diameter silicone air line from a standard aquarium pump and permeated through an air stone. The temperature of the room was maintained between 18 °C and 20 °C.

**Table 3:** Summary of nested experimental design. Each treatment had six replicates.

*Treatment Number*	*Reservoir Water (ml)*	*Chlorella Stock (ml)*	*Synechocystis Stock (ml)*	*Number of Mussels*	*Phosphate Stock (ml)*
1	225	25	0	2	0
2	225	25	0	0	0
3	225	0	25	2	0
4	225	0	25	0	0
5	200	25	25	2	0
6	200	25	25	0	0
7	225	25	0	2	2.5
8	225	25	0	0	2.5
9	225	0	25	2	2.5
10	225	0	25	0	2.5
11	200	25	25	2	2.5
12	200	25	25	0	2.5

Experiments used a nested design with six replicates per treatment. Treatments monitored the growth of *Chlorella* alone, *Synechocystis* alone, or *Chlorella* + *Synechocystis* in co-culture. Experiments were run in the presence and absence of mussels, and in low and high phosphorus conditions, such that a total of 72 experimental were undertaken (Table 3).

Mussel treatments each used two zebra mussels, which increases the chances of a constant grazing pressure on the algae. Zebra mussels of the size used in this experiment (35 mm ± 5SE) have been shown to have clearance rates of 247 ml∙mussel^−1^∙hour^−1^^57^ and even as high as 574 ml∙mussel^−1^∙hour^−1^^8^. Therefore, a stocking density of two mussels should result in the equivalent total volume of the experiment being filtered up to twice every hour, allowing for a shorter experimental run time. The mussels expressed a constant feeding pattern following the algae inoculation, with at least one of the mussels feeding at any one time.

Low phosphorus treatments used water from the collection site without enrichment. High phosphorus treatments were enriched with a phosphate stock solution containing 870 µg/L K_2_HPO_4_ and 680 µg/L KH_2_PO_4_.The concentration of orthophosphate (PO_4_-P) in high P and low P treatments was calculated using a phosphate cuvette test (Hach Lange LCK349 Phosphate (Ortho/Total) cuvette test 0.05–1.5 mg/L PO4-P). The test indicated the low P treatments contained <3 µg PO_4_-P/L, whereas the high P treatments contained 412 µg PO_4_-P/L.

A calibrated fluoroprobe (bbe Moldaenke GmbH, Schwentinental, Germany) was used to estimate the number of *Chlorella* and *Synechocystis* cells present in each sample (cells/ml). The fluoroprobe discriminated between *Chlorella* and *Synechocystis* by measuring the presence of photosynthetic accessory pigments, which result in identifiable ‘fluorescence fingerprints’. *Chlorella* is rich in chlorophyll *a* and *b*, while *Synechocystis* contains phycocyanin. The fluoroprobe measured the fluorescence emitted by the sample following excitation of these different photosynthetic accessory pigments using six LEDs emitting at different wavelengths (370 nm, 470 nm, 525 nm, 570 nm, 590 nm and 610 nm)^58^. The fluoroprobe was mounted in the ‘workstation 25 standard version’ in which it can analyse the contents of a 25 ml cuvette. The bbe++ software package^59^ was used to set the measurement parameters, operate the fluoroprobe and log the results of the experiments.

The measurements were carried out between the 27 and 30 March 2018. Samples from each aquarium were taken at 10 intervals over 84 hours. A 25 ml sample was collected from each aquarium, after resuspending settled algae, at each time point using a syringe then added to the fluoroprobe cuvette for measurement. After the measurement was taken the sample was replaced in the aquarium. The cuvette was rinsed with DI water and dried between each measurement.

### Statistical analysis

Two generalised linear mixed models (GLMM), with a Poisson log link and error distribution, were used to identify relationships between the number of algae cells/ml and the set variations in conditions. The fixed effects for assessing changes in cells/ml for *Chlorella* and *Synechocystis* across all treatments were P level (high or low), mussels (presence or absence), treatment type (Single algae culture or mixed culture) and the interaction between the mussels and treatment type. The random effects were the number of hours and the individual experiments. This accounts for the modelling of potentially differing intercept values and curves between experiments. Analysis was performed in R Studio (v.1.0.136)^60^ with the package lme4 (v1.1-12)^61^.

## Supplementary information

R code for Figure 1 and GLMM

Dataset 1

## Data Availability

Cell count data, raw figures, tables, and R code to reproduce figures and the Generalised Linear Mixed Model results are available in the supplementary information.
