# Supplementary material for: Harnessing Synthetic Ecology for commercial algae production
Source: Sci Rep. 2019 Jul 5;9:9756. doi: 10.1038/s41598-019-46135-6 (PMC6611825; doi:10.1038/s41598-019-46135-6)
Supplement: Supplementary file 1 — R code for Figure 1 and GLMM [file 41598_2019_46135_MOESM1_ESM.pdf]

Harnessing Synthetic Ecology for commercial algae production

Sam A. Reynolds[1*], Matthew P. Davey[2], David C. Aldridge[1]

1. Department of Zoology, University of Cambridge, The David Attenborough Building, Pembroke Street Cambridge CB2 3QZ, UK.

2. Department of Plant Sciences, University of Cambridge, Downing Street, Cambridge CB2 3EA, UK

*Corresponding author: sar87@cam.ac.uk, (+44) 07846003387

Figure 1a and 1b R Code

```
#Select the Supplementary Data File

data1<-read.csv(file.choose())

#Splitting the dataset by Mussels and No Mussels

Mdata<-data1[data1$Mussel=="Mussel",]

NMdata<-data1[data1$Mussel=="No Mussel",]

#Splitting the dataset by Low and High P

HPdata<-data1[data1$P=="High P",]

LPdata<-data1[data1$P=="Low P",]

# s.d and mean function data frame creation

library(tidyr)
library(ggplot2)
library(lsmeans)
library(splines)

summarySE <- function(data=NULL, measurevar, groupvars=NULL, na.rm=FALSE,
                      conf.interval=.95, .drop=TRUE) {
  library(plyr)

  # New version of length which can handle NA's: if na.rm==T, don't count them
  length2 <- function (x, na.rm=FALSE) {
    if (na.rm) sum(!is.na(x))
    else       length(x)
  }

  # This does the summary. For each group's data frame, return a vector with
  # N, mean, and sd
  datac <- ddply(data, groupvars, .drop=.drop,
                 .fun = function(xx, col) {
                   c(N    = length2(xx[[col]], na.rm=na.rm),
                     mean = mean   (xx[[col]], na.rm=na.rm),
```

```
                    sd   = sd     (xx[[col]], na.rm=na.rm)
                  )
                },
                measurevar
  )

  # Rename the "mean" column
  datac <- rename(datac, c("mean" = measurevar))

  datac$se <- datac$sd / sqrt(datac$N)  # Calculate standard error of the mean

  # Confidence interval multiplier for standard error
  # Calculate t-statistic for confidence interval:
  # e.g., if conf.interval is .95, use .975 (above/below), and use df=N-1
  ciMult <- qt(conf.interval/2 + .5, datac$N-1)
  datac$ci <- datac$se * ciMult

  return(datac)
}

#Figure 1a Plot (High P)

High_P <- summarySE(HPdata, measurevar="Cells.ml.10000",
groupvars=c("No.Hours","Algae","Treatment.Type","Mussel"))

pd <- position_dodge(0.5) # move them .05 to the left and right

High_Pplot <- ggplot(data = High_P, aes(x = No.Hours, y = Cells.ml.10000, color =
Algae, shape = Algae, linetype = Algae)) +
  geom_line(position = pd, size =1) +
  geom_errorbar(aes(ymin = Cells.ml.10000 - se, ymax = Cells.ml.10000 + se), width
= 2, position = pd) +
  geom_point(position = pd, size=2)+
  facet_grid(Mussel~Treatment.Type)+
  ggtitle ("(a) High P Conditions")+
  labs(x="Number of Hours", y="Cells per ml (x10000)")+
  theme_bw()+
  theme(legend.position = "right",
        plot.title = element_text(size=20, hjust = 0.0, vjust=2.12, face
="italic"),
        strip.text.x = element_text(size = 15),
        strip.text.y = element_text(size = 15),
        axis.title = element_text(size = 15),
```

```
        legend.text = element_text(size = 20),
        legend.title = element_text(size = 20))

print (High_Pplot + scale_colour_manual(values = c("dodgerblue", "violetred")))
```

#Figure 1b plot (Low P)

```
Low_P <- summarySE(LPdata, measurevar="Cells.ml.10000",
groupvars=c("No.Hours","Algae","Treatment.Type","Mussel"))

pd <- position_dodge(0.5) # move them .05 to the left and right

Low_Pplot <- ggplot(data = Low_P, aes(x = No.Hours, y = Cells.ml.10000, color =
Algae, shape = Algae, linetype = Algae)) +
  geom_line(position = pd, size =1) +
  geom_errorbar(aes(ymin = Cells.ml.10000 - se, ymax = Cells.ml.10000 + se), width
= 2, position = pd) +
  geom_point(position = pd, size=2)+
  facet_grid(Mussel~Treatment.Type)+
  ggtitle ("(b) Low P Conditions")+
  labs(x="Number of Hours", y="Cells per ml (x10000)")+
  theme_bw()+
  theme(legend.position = "right",
        plot.title = element_text(size=20, hjust = 0.0, vjust=2.12, face
="italic"),
        strip.text.x = element_text(size = 15),
        strip.text.y = element_text(size = 15),
        axis.title = element_text(size = 15),
        legend.text = element_text(size = 20),
        legend.title = element_text(size = 20))

print (Low_Pplot + scale_colour_manual(values = c("dodgerblue", "violetred")))
```

Generalised Linear Mixed Model R Code

```
# Inputting the data and adding the data levels, Select the Supplementary Data File

test.data <- read.csv(file.choose())

test.data$P <- factor(test.data$P, levels = c("Low P", "High P"))
test.data$Mussel <- factor(test.data$Mussel, levels = c("No Mussel", "Mussel"))

install.packages("lme4")
library(lme4)

# Chlorella Data

C.data <- subset(test.data, test.data$Algae == "C")
C.data$Treatment.Type<-factor(C.data$Treatment.Type, levels = c("Chlorella",
"Mixed"))
C.data$No.Hours <- as.factor(C.data$No.Hours) #makes hours a factor rather than
continious variable
test.glmmC <- glmer(Cells.ml ~ 1 + P + Mussel*Treatment.Type + (1|No.Hours) +
(1|Bucket.Number),
                    data = C.data, family = poisson(link = "log"))

summary(test.glmmC)
plot(test.glmmC)

# Synechocystis Data

S.data <- subset(test.data, test.data$Algae == "S")
S.data$Treatment.Type<-factor(S.data$Treatment.Type, levels = c("Synechocystis",
"Mixed"))
S.data$No.Hours <- as.factor(S.data$No.Hours) #makes hours a factor rather than
continious variable
test.glmmS <- glmer(Cells.ml ~ 1 + P + Mussel*Treatment.Type + (1|No.Hours) +
(1|Bucket.Number),
                    data = S.data, family = poisson(link = "log"))

summary(test.glmmS)
plot(test.glmmS)
```
